# Protection of *Sinorhizobium* against Host Cysteine-Rich Antimicrobial Peptides Is Critical for Symbiosis

**DOI:** 10.1371/journal.pbio.1001169

**Published:** 2011-10-04

**Authors:** Andreas F. Haag, Mikhail Baloban, Monica Sani, Bernhard Kerscher, Olivier Pierre, Attila Farkas, Renato Longhi, Eric Boncompagni, Didier Hérouart, Sergio Dall’Angelo, Eva Kondorosi, Matteo Zanda, Peter Mergaert, Gail P. Ferguson

**Affiliations:** 1 School of Medicine & Dentistry, Institute of Medical Sciences, University of Aberdeen, Aberdeen, United Kingdom; 2 Institut des Sciences du Végétal, Centre National de la Recherche Scientifique, Gif-sur-Yvette, France; 3 Consiglio Nazionale delle Ricerche–Istituto di Chimica del Riconoscimento Molecolare C.N.R.-I.C.R.M., Milano, Italy; 4 KemoTech s.r.l., Pula, Italy; 5 Interactions Biotiques et Santé Végétale, Institut National de la Recherche Agronomique, Centre National de la Recherche Scientifique, Université de Nice–Sophia Antipolis, Sophia-Antipolis, France; 6 Institute for Plant Genomics, Human Biotechnology and Bioenergy, Bay Zoltan Foundation for Applied Research, Szeged, Hungary; 7 School of Medical Sciences, Institute of Medical Sciences, University of Aberdeen, Aberdeen, United Kingdom; 8 Biological Research Centre, Hungarian Academy of Sciences, Szeged, Hungary; The University of North Carolina at Chapel Hill, United States of America

## Abstract

*Sinorhizobium meliloti* differentiates into persisting, nitrogen-fixing bacteroids within root nodules of the legume *Medicago truncatula*. Nodule-specific cysteine-rich antimicrobial peptides (NCR AMPs) and the bacterial BacA protein are essential for bacteroid development. However, the bacterial factors central to the NCR AMP response and the *in planta* role of BacA are unknown. We investigated the hypothesis that BacA is critical for the bacterial response towards NCR AMPs. We found that BacA was not essential for NCR AMPs to induce features of *S. meliloti* bacteroids in vitro. Instead, BacA was critical to reduce the amount of NCR AMP-induced membrane permeabilization and bacterial killing in vitro. Within *M. truncatula*, both wild-type and BacA-deficient mutant bacteria were challenged with NCR AMPs, but this resulted in persistence of the wild-type bacteria and rapid cell death of the mutant bacteria. In contrast, BacA was dispensable for bacterial survival in an *M. truncatula dnf1* mutant defective in NCR AMP transport to the bacterial compartment. Therefore, BacA is critical for the legume symbiosis by protecting *S. meliloti* against the bactericidal effects of NCR AMPs. Host AMPs are ubiquitous in nature and BacA proteins are essential for other chronic host infections by symbiotic and pathogenic bacteria. Hence, our findings suggest that BacA-mediated protection of bacteria against host AMPs is a critical stage in the establishment of different prolonged host infections.

## Abstract 2

A bacterial membrane protein, BacA, protects *Sinorhizobium meliloti* against the antimicrobial activity of host peptides, enabling the peptides to induce bacterial persistence rather than bacterial death.

## Author Summary

Certain bacterial species have the unique capacity to enter into eukaryotic host cells and establish prolonged infections, which can be beneficial (e.g. bacterial-legume symbiosis) or detrimental (e.g. chronic disease) for the host. However, the mechanisms by which bacteria persist in host cells are poorly understood. Legume peptides and the bacterial BacA membrane protein play essential roles in enabling bacteria to establish prolonged legume infections. However, the biological function of BacA in persistent legume infections has eluded scientists for nearly two decades. In this article, we investigated a potential relationship between legume peptides and BacA in the establishment of prolonged bacterial-legume infections. We found that BacA was critical to protect bacteria against the antimicrobial action of legume peptides, thereby allowing the peptides to induce bacterial persistence within the legume rather than rapid bacterial death. Mammalian hosts also produce peptides in response to invading microorganisms and BacA proteins are critical for medically important bacterial pathogens such as *Mycobacterium tuberculosis* to form prolonged mammalian infections. Therefore, our results suggest that BacA-mediated protection against host peptides might be a conserved mechanism used by both symbiotic and pathogenic bacterial species to establish long-term host infections.

## Introduction

The alpha-proteobacterium *Sinorhizobium meliloti* forms a symbiosis within specialized root organs, called nodules with *Medicago* species of leguminous plants, including the agriculturally important alfalfa and the model legume *Medicago truncatula* [1]. The bacteria induce the organogenesis of nodules and infect the host plant via tubular structures called infection threads from which they are delivered into the nodule cells by an endocytosis-like process. The internalized bacteria are encapsulated within a plant-derived membrane, forming an organelle-like symbiosome compartment [1]. Within the symbiosome, *S. meliloti* differentiates into nitrogen-fixing bacteroids, which persist resulting in a chronic intracellular infection and symbiosis [2]. *S. meliloti* bacteroids formed within *M. truncatula* have elongated morphologies, undergo extensive genome amplification, have a decreased cytoplasmic membrane integrity, and lack reproductive capacity (i.e., terminal differentiation) relative to their free-living, bacterial form [3]. Bacteroid differentiation is mediated within the host compartments by a large family of several hundred legume nodule-specific cysteine-rich (NCR) peptides, which are transported into these compartments [4]. A subset of NCR peptides (here called NCR AMPs) exhibits antimicrobial activity in vitro [4]. NCR AMPs are structurally and functionally similar to plant and mammalian defensins, as they have conserved cysteine residues, which are predicted to form defined disulfide (S-S) bridges, and are cationic [5]. Defensins are a widespread class of AMPs, which are key effectors of innate immunity in both animals and plants [6],[7].

Despite knowledge of the critical role of *Medicago* NCR AMPs in mediating bacteroid development, the bacterial factors central to this response are unknown. Nearly two decades ago, the *S. meliloti* cytoplasmic membrane BacA protein was discovered to be essential for bacteroid development within *Medicago* legumes [8]. *S. meliloti* BacA-deficient mutant bacteria infect nodule cells and enter into the host symbiosome compartments but were unable to develop into the typical elongated bacteroids, which persist [8],[9]. Mammalian pathogens such as *Brucella abortus* and *Mycobacterium tuberculosis* also have BacA homologs, which were found to be critical for prolonged murine infections [10],[11]. Therefore, BacA proteins are essential for bacterial symbionts and pathogens to establish prolonged intracellular infections of plants and mammals, respectively.

The precise in vivo function of BacA proteins is unknown. *S. meliloti*, *B. abortus*, and *M. tuberculosis* produce unusual fatty acid-modified lipids [10],[12],[13]. BacA proteins of *S. meliloti* and bacterial pathogens are involved in the modification of lipids with these unusual fatty acids in vitro [12]. However, these unusual lipid modifications were found to be important but not essential for *S. meliloti* bacteroid development during the legume symbiosis [14]. It was recently discovered that BacA proteins are critical for rhizobial bacteroid development in other legume hosts producing NCR peptides such as *Pisum sativum* (pea) and *Astragalus sinicus*, but they are dispensable for this process in *Phaseolus vulgaris* (bean), *Vigna unguiculata* (cowpea), and *Lotus japonicus*, which are legume hosts naturally devoid in these peptides (Table S1) [15]–[19]. The presence of NCR peptides and the requirement of BacA for an effective symbiosis also correlate with the formation of elongated, terminal bacteroids in nodules. In contrast, bacteroids are morphologically undifferentiated and can de-differentiate (i.e., reversible differentiation) in legumes that lack NCR peptides and do not require a functional BacA for symbiosis (Table S1) [3],[5],[20]. Since mammalian pathogens also encounter host AMPs early in their infection process, in the extracellular environment, as well as in their intracellular niche [6],[7], we hypothesized that BacA proteins are critical for chronic intracellular infections by playing a central role in the bacterial response towards host AMPs. In this study, we found that the BacA protein is essential for protection of *S. meliloti* both in vitro and *in planta* against the antimicrobial activities of host NCR peptides. Since BacA proteins are found in diverse bacterial species [8],[10],[11] and AMPs are ubiquitous in nature [7], these findings have important implications for the molecular mechanisms of other chronic bacterial-host interactions.

## Results

### A Specifically Folded NCR Peptide Induces *S. meliloti* Bacteroid Features In Vitro

**Figure 1:**
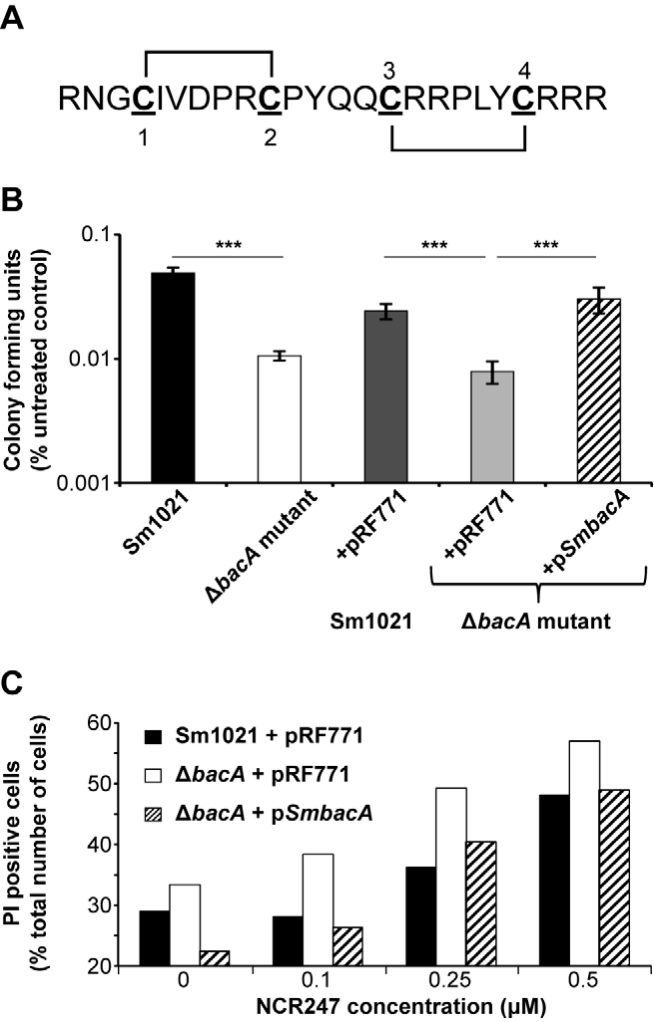
BacA proteins protect *S. meliloti* in vitro against the antimicrobial activity of NCR247. (A) NCR247 peptide sequence showing the two S-S bridges between cysteine residues 1 and 2 and 3 and 4. (B) Colony forming ability of the indicated strains was assessed after exposure towards 20 µM of the NCR247 peptide for 3 h. (C) The PI fluorescence of cells was determined by flow cytometry after treatment of cultures with and without the NCR247 peptide for 3 h. Bars represent mean ± SD. The significance value ***p≤0.001 was determined using ANOVA followed by a Student Newman-Keuls post-test and results are representative for at least two independent experiments.

To enable us to investigate a potential role for BacA in the bacterial response towards NCR AMPs, we synthesized the *M. truncatula* NCR247 peptide (Figure 1A; Figure S1A,B) using a procedure [21] which protects the sulphydryl groups of cysteine-rich peptides during their synthesis preventing inappropriate inter-peptide disulphide (S-S) bridge formation. Using Chou-Fasman rules [22], we predicted the most likely S-S bridges of NCR247 were between cysteines 1 and 2 and cysteines 3 and 4 (Figure 1A) and thus synthesized this folded state of NCR247 through selective de-protection of the sulphydryl groups. The synthesized NCR247 peptide had a mass of 3004.5 Da, confirming two S-S bridges, and a purity of greater than 95% (w/v) (Figure S1A,B).

Treatment of the *S. meliloti* wild-type strain in vitro with 2 or 4 µM NCR247 induced cell elongation (Figures S2A,C) and genomic DNA amplification (Figure S2B,D), which are features of *in planta S. meliloti* bacteroids. When *S. meliloti* differentiates into a bacteroid, it increases in length approximately 5–10-fold and has a 12–24-fold increase in its genomic DNA content [3]. We found that, although there was variation in the response of individual *S. meliloti* cells within the population towards NCR247 exposure, a large proportion of the cells showed a considerable increase in their forward scatter (a measure for cell size) and contained up to 16 copies of their genomic DNA content after treatment with 4 µM NCR247 (Figure S2C and D, respectively). However, no difference was observed in the ability of the same concentrations of NCR247 to induce these bacteroid features in the *S. meliloti* BacA-deficient mutant in vitro (Figure S2E to H). Therefore, NCR247 was sufficient in itself to induce features of *in planta S. meliloti* bacteroids, but BacA function was not critical for this process, despite the inability of the BacA-deficient mutant to form bacteroids *in planta* [8],[9].

### BacA Proteins Protect *S. meliloti* Against the Antimicrobial Activity of NCR Peptides

A subset of NCR peptides possesses antimicrobial activity in vitro and reduces the colony forming ability of *S. meliloti* [4]. Hence, treatment of wild-type *S. meliloti* with 20 µM NCR247 reduced its subsequent colony forming ability (Figure 1B). The 2–4 µM concentrations were found to be sub-lethal (unpublished data), indicating that the effects on cell size and DNA content at low NCR247 concentrations were uncoupled from the killing, which is only observed at higher concentrations. Importantly, an *S. meliloti* BacA-deficient mutant was more sensitive towards the antimicrobial action of NCR247 in vitro (Figure 1B), showing that BacA was critical to prevent hypersensitivity of *S. meliloti* towards this host peptide. The plasmid-encoded wild-type *S. meliloti bacA* gene (p*SmbacA*) restored the colony forming ability of the BacA-deficient mutant after exposure to NCR247 (Figure 1B), confirming that BacA mediated protection of *S. meliloti* against NCR247 in vitro. NCR peptides reduce the colony forming ability of *S. meliloti* in vitro by lowering the integrity of the cytoplasmic membrane, resulting in the entry of the usually non-membrane permeable fluorescent dye, propidium iodide (PI) [4]. We found that NCR247 treatment increased the PI fluorescence of the BacA-deficient mutant to a greater extent than the wild-type strain and the presence of p*SmbacA* restored the PI fluorescence of the BacA-deficient mutant to that of the wild-type strain with control plasmid in the presence of NCR247 (Figure 1C). These findings demonstrated that the hypersensitivity of the *S. meliloti* BacA-deficient mutant towards the NCR247 peptide in vitro was due to an increase in NCR247-induced loss of cytoplasmic membrane integrity. We also found that the *S. meliloti* BacA-deficient mutant was hypersensitive to another *M. truncatula* NCR AMP, NCR035, in vitro relative to the wild-type strain (Figure S3A,B). Taken together, these findings showed that BacA protected *S. meliloti* against the antimicrobial activity of different *M. truncatula* NCR AMPs in vitro.

The *B. abortus* BacA protein is essential for chronic intracellular mammalian infections [11]. A plasmid-encoded *B. abortus* wild-type *bacA* gene (p*BabacA*) complements the chronic infection defect of an *S. meliloti* BacA-deficient mutant in *Medicago* species of legumes [23]. We also found that p*BabacA* protected the *S. meliloti* BacA-deficient mutant strain to a similar extent as the wild-type *S. meliloti bacA* gene against the antimicrobial action of NCR247 in vitro (Figure S4). These findings provide further evidence that the *S. meliloti* and *B. abortus* BacA proteins are functional homologs and demonstrated that *B. abortus* BacA protected bacteria against the antimicrobial activity of a defensin-like AMP.

### An *S. meliloti* BacA-Deficient Mutant Is Challenged with NCRs In Vivo

The hypersensitivity of the BacA-deficient mutant to NCRs in vitro suggested a scenario for the inability of this strain to develop into bacteroids within the legume. Upon entry into the host compartment, *S. meliloti* is confronted with NCRs leading to bacteroid differentiation in the case of a wild-type strain, which has a sufficient level of resistance to NCRs. In contrast, the BacA-deficient mutant would be killed by NCR AMP challenge, preventing bacteroid differentiation. This scenario required that NCRs be transported into the symbiosome containing the BacA-deficient mutant as well. Using RT-PCR in combination with *NCR* specific primers, we analyzed *NCR* gene expression in roots and nodules induced by wild-type *S. meliloti* and a BacA-deficient mutant strain (Figure S5). *NCR084*, *NCR247*, and *NCR035* were expressed to similar levels in nodules induced by both strains while *NCR001* was only weakly expressed in the nodules of the BacA-deficient mutant. This is in agreement with the previously described two consecutive transcriptome waves that characterize the transcriptional changes during nodule development [9]. *NCR084*, *NCR247*, and *NCR035* are induced during the first wave, which is still activated in nodules of the BacA-deficient mutant while *NCR001* is expressed during the second transcriptome switch, which is not activated anymore in nodules of the BacA-deficient mutant [9]. Thus, at least a subset of *NCR* genes is still expressed in nodules of the BacA-deficient mutant.

**Figure 2:**
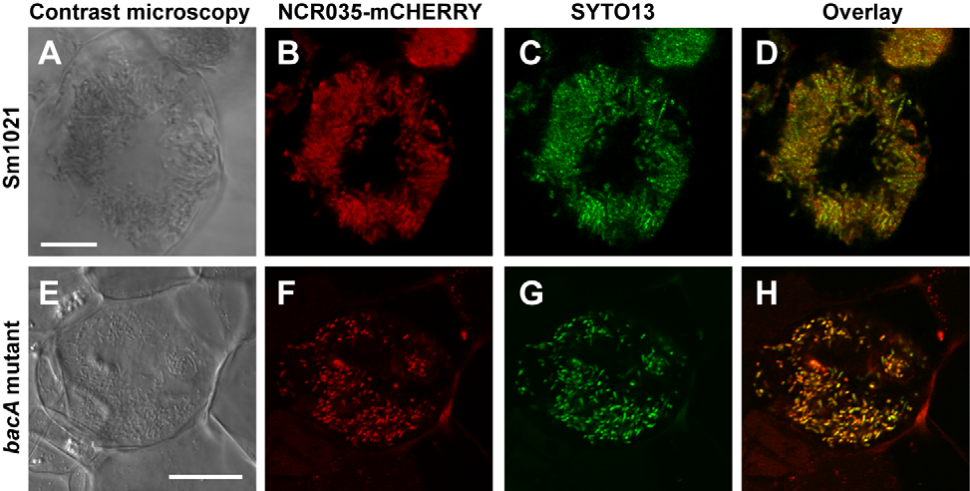
The *S. meliloti bacA* mutant is challenged with NCR peptides. Microscopy of transgenic *M. truncatula* nodule cells expressing NCR035-mCHERRY under the control of the *NCR035* promoter infected with either the *S. meliloti* wild-type (A–D) or BacA-deficient mutant (E–H) strains. Differential Interference Contrast microscopy (A,E), confocal microscopy of NCR035-mCHERRY localization (red) (B and F), bacterial localization with the DNA stain SYTO13 (green) (C,G), and an overlay of NCR035-mCHERRY and SYTO13 localization (D,H). In both the wild-type and BacA-deficient mutant infected nodules, NCR035 co-localizes with the bacteria. Scale bars are 10 µm.

To test whether NCRs were also transported to symbiosomes, transgenic *M. truncatula* roots expressing translational fusions of either the NCR035 or NCR084 peptide with the mCHERRY red fluorescent protein, under the control of their own promoter, were inoculated with either the *S. meliloti* wild-type or BacA-deficient mutant. In agreement with our previous results [4], the fluorescently labelled NCR peptides co-localized entirely with the wild-type strain bacteroids (Figure 2A–D), consistent with their trafficking to the bacterial compartment. Likewise, we found that the NCRs also co-localized with the BacA-deficient mutant-bacteria within the symbiosomes (Figure 2E–H), demonstrating that the mutant bacteria were challenged with NCRs within their host compartment. However, in contrast to the wild-type strain-infected nodules, a proportion of the NCR peptides were secreted to the exterior of the plant cell in nodules infected with the BacA-deficient mutant (Figure S6). Combined, these findings showed that BacA is important for NCR peptide trafficking to the symbiosomes but also demonstrated that an *S. meliloti* BacA-deficient mutant is challenged with NCR peptides in vivo.

### BacA Protects *S. meliloti* from Rapid Cell Death upon Entry Into the Host Compartment

**Figure 3:**
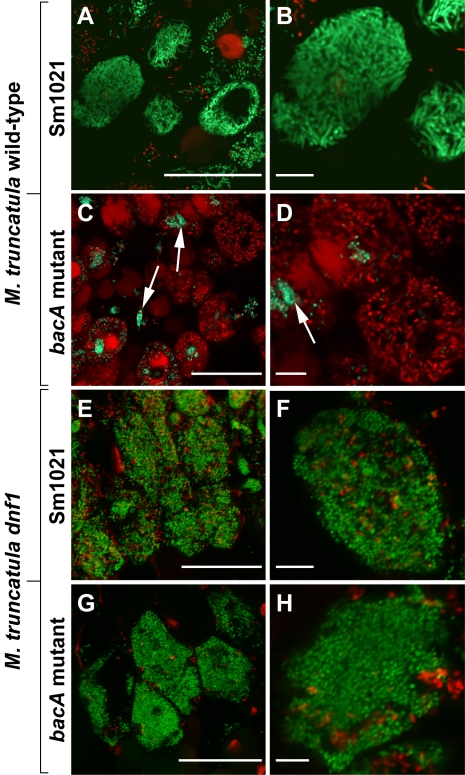
The *S. meliloti* BacA-deficient mutant undergoes rapid death in symbiosomes. Confocal microscopy of infected cells in nodules of wild-type *M. truncatula* (A–D) or the *M. truncatula dnf1* mutant (E–H). Nodules were infected by either the *S. meliloti* Sm1021 wild-type strain (A,B,E,F) or the BacA-deficient mutant (C,D,G,H). Nodules were stained with a mixture of SYTO9 (green signal) and PI (red signal). Live bacterial cells are stained by SYTO9 and dead bacteria are stained by PI. Arrows in (C,D) indicate rhizobia in infection threads. Scale bars are 50 µm (A,C,E,G) or 10 µm (B,D,F,H).

Microscopy of root nodules induced by the *S. meliloti* BacA-deficient mutant showed that it was specifically defective in bacteroid development within the symbiosome compartments [8]. To more specifically investigate bacterial viability during the host interaction, we used a live/dead staining procedure involving a mixture of the nucleic acid fluorescent dyes, SYTO9 and PI, which were applied to legume nodule sections. In the presence of both dyes, live bacterial cells with intact cytoplasmic membranes are stained by SYTO9 and fluoresce green while dead bacteria with damaged cytoplasmic membranes are stained by PI and fluoresce red [24]. Using this procedure, we found that as expected the wild-type bacteria fluoresced green within the host compartments, indicating they were viable and metabolically active (Figure 3A,B; Figure S7A). In contrast, although the BacA-deficient mutant in the infection threads, which deliver the bacteria into the host cell compartment [2], fluoresced green (Figure 3C,D, arrows), the vast majority of mutant bacteria fluoresced red upon their release into the host compartments (Figure 3C,D; Figure S7B). Thus, these findings showed that the BacA-deficient mutant bacteria are rapidly killed upon their uptake into the host compartment. They were also consistent with our in vitro findings demonstrating that BacA is critical to prevent the hypersensitivity of *S. meliloti* towards the antimicrobial activity of host NCRs.

### BacA Is Dispensable for Bacterial Survival in a Legume Mutant Defective in NCR Trafficking

To provide further evidence in support of our hypothesis that the rapid death of the BacA-deficient bacteria within the host compartment was the result of NCR peptide challenge, we took advantage of the *M. truncatula dnf1* mutant, which is defective in a nodule-specific signal peptidase complex involved in the trafficking of NCR peptides [25]. As a consequence, the transport of NCRs into the symbiosomes is blocked in the *M. truncatula dnf1* mutant [4]. Since NCR peptide challenge is essential for *S. meliloti* to differentiate into persisting, nitrogen-fixing bacteroids, the *dnf1* mutant nodules become infected by *S. meliloti* bacteria, which are unable to differentiate into bacteroids [4]. Applying the live/dead staining procedure, we found that the *S. meliloti* wild-type strain fluoresced green within the symbiosomes of *dnf1* mutant nodules, indicating that they were healthy despite the absence of bacteroid differentiation (Figure 3E,F; Figure S7C). In fact, the bacteria remained healthy for an extended time, within several layers of symbiotic cells, and only died in the oldest, root-proximal symbiotic cells of the *dnf1* mutant nodules as revealed by the red PI staining (Figure S8). More importantly, we also found that, unlike in the host compartment of wild-type *M. truncatula* (Figure 3C,D; Figure S7B), the released bacteria of the BacA-deficient mutant fluoresced green within the symbiosome compartments of the *M. truncatula dnf1* mutant (Figure 3G,H; Figure S7D; compare with Figure 3C,D; Figure S7B). Consequently, these findings showed that in the absence of NCR challenge within the host compartment, the BacA-deficient mutant remained viable instead of being rapidly killed.

## Discussion

**Figure 4:**
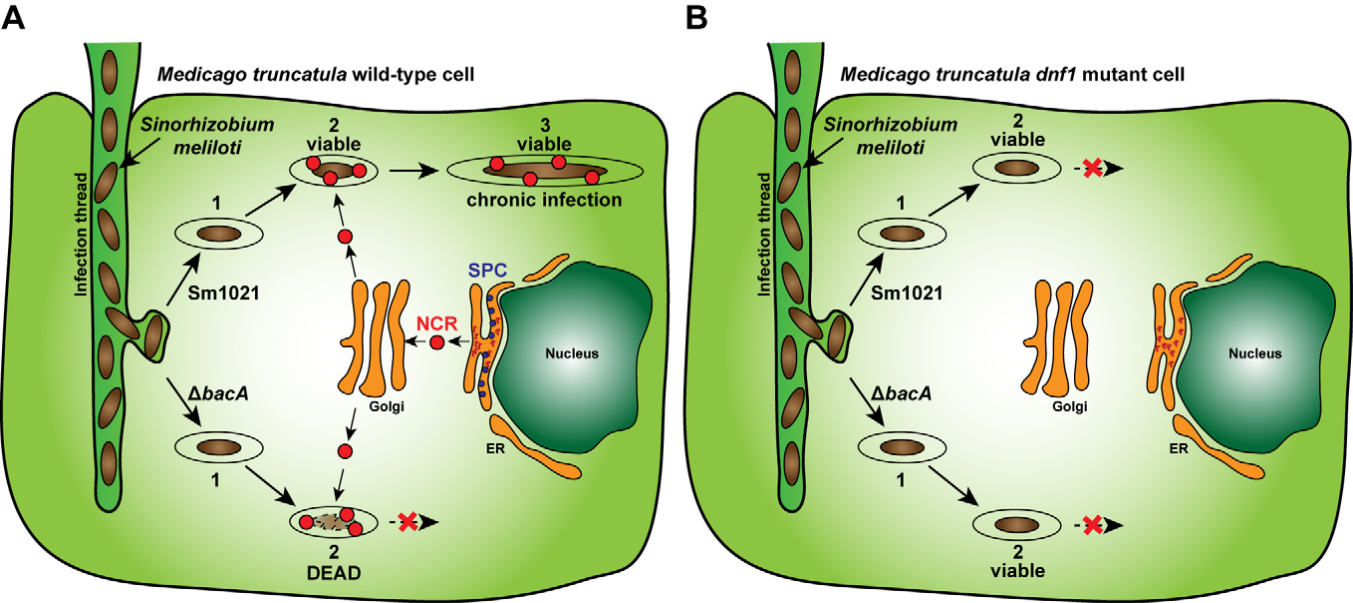
Hypersensitivity towards host AMPs prevents chronic bacterial infections. (A) In a wild-type *M. truncatula* nodule cell, the *S. meliloti* wild-type strain (Sm1021) and BacA-deficient mutant (Δ*bacA*) bacteria are released from the infection threads into the membrane-bound symbiosome (1). The bacteria are then challenged with host NCR AMPs (2), which mediate the differentiation of the wild-type strain into a nitrogen-fixing bacteroid, resulting in the chronic infection (3). In contrast, the BacA-deficient mutant is hypersensitive towards the host NCR AMPs and is killed by them rather than forming the chronic infection (2). (B) The *M. truncatula dnf1* mutant is defective in a nodule-specific signal-peptidase complex (SPC) and as a result the NCR peptides are no longer transported to the bacterial compartment but are retained in the endoplasmic reticulum (ER) [4]. The *S. meliloti* wild-type strain and BacA-deficient mutant enter into the symbiosomes (1) and remain viable due to the absence of NCR peptide challenge (2). However, neither strain can differentiate into N_2_-fixing bacteroids due to the absence of NCRs.

In this study, we showed that (i) the cytoplasmic membrane protein BacA protects *S. meliloti* from being hypersensitive to NCR AMPs in vitro by reducing the degree of peptide-induced inner membrane permeabilization, (ii) BacA is essential for survival of *S. meliloti* in wild-type *M. truncatula* symbiosomes, and (iii) BacA is dispensable for *S. meliloti* symbiosome survival in an *M. truncatula dnf1* mutant legume, which is defective in trafficking NCR peptides to the symbiosome compartment. Therefore, our findings have shown that there is a strong correlation between the presence of symbiosome NCR AMPs and the requirement of BacA for *S. meliloti* symbiosome survival (Figure 4). These findings are also consistent with previous studies whereby BacA proteins were found only to be critical for the rhizobium-legume symbiosis in hosts such as *Medicago*, peas, and *Astragalus*, which produce NCR AMPs, but non-essential in legumes naturally devoid of NCR AMPs such as bean, cowpea, or *Lotus* (Table S1). Based on these findings, we propose a model whereby BacA is critical to minimize the antibacterial action of NCR AMPs on *S. meliloti*, thereby enabling proper bacteroid development. Since the function of bacterial BacA proteins in prolonged plant and mammalian infections have eluded investigators for many years [8],[10],[11], this study represents a significant advance in understanding the in vivo role of these proteins.

We found that the difference between the wild-type and BacA-deficient strain in their response to the in vitro treatments was relatively modest compared to the *in planta* observations, where the difference in viability between both strains was substantial. However, a major difference between the in vitro and *in planta* conditions is that a highly complex cocktail of several hundred different NCR AMPs are produced *in planta* [5]. In addition, the environmental parameters within the nodule such as microaerobic conditions and low pH [1] might also impact the effect of NCRs on *S. meliloti*.

Although the BacA-deficient mutant bacteria were challenged with NCR peptides in vivo, a significant proportion of these peptides were transported to the plant cell surface rather than to the *S. meliloti* BacA-deficient-containing symbiosome compartment. Since the BacA-deficient mutant bacteria are killed within the symbiosomes [8], a reduced amount of target symbiosome compartments for NCR transport could lead to secretion of excess NCR peptides to the exterior of the plant cell. Further studies investigating NCR peptide trafficking with other *S. meliloti* mutants defective in bacteroid development would be necessary to confirm that this is the case.

Our data showed that the *S. meliloti* BacA protein is critical to reduce the amount of cytoplasmic membrane damage as a result of NCR AMP challenge in vitro. At present, we do not know the precise mechanism by which BacA limits NCR-induced cytoplasmic membrane damage. An *S. meliloti* BacA-deficient mutant has ∼50% reduction in the content of an unusual outer membrane lipid relative to the wild-type strain [12],[26]. Since this unusual lipid is important in outer membrane stability [27], one possibility is that NCR AMPs are more effective at disrupting the outer membrane of the *S. meliloti* BacA-deficient mutant relative to the wild-type strain leading to the increased damage to the inner membrane. However, an *S. meliloti* mutant, which completely lacks this unusual lipid, still forms persistent legume infections and can survive in NCR AMP-containing symbiosomes [14],[27], suggesting that BacA must have an additional role in the response of *S. meliloti* towards host NCRs. Since an *S. meliloti acpXL* mutant, which is completely defective in the biosynthesis of the unusual lipid A [14],[27], was osmotically unstable and lysed in the low osmolarity buffer used to test NCR peptide sensitivity in vitro (unpublished data), we were unable to determine the role of this unusual lipid in the bacterial response to the NCR AMPs. Moreover, BacA could affect the bacterial envelope in other ways, for example in its protein composition as suggested by transcriptome analysis in the *Rhizobium leguminosarum bacA* mutant [15], which in turn may be related to the hypersensitivity towards NCR peptides in the *bacA* mutants. In addition, BacA is predicted to be a cytoplasmic membrane subunit of an ABC transporter and our previous studies found that it plays a key role in the uptake of structurally diverse, non-membrane disrupting peptides in *S. meliloti* in vitro [15],[28], suggesting that BacA may be a peptide transporter. At present it is not known if BacA is directly involved in peptide transport in bacteria or whether it indirectly influences the uptake of those peptides. If BacA is directly involved in peptide transport, BacA-mediated uptake of NCR peptides might reduce their ability to induce membrane damage. Alternatively, BacA might confer protection by being involved in the efflux of NCR peptides. However, further studies will be necessary to understand whether NCR AMPs have intracellular targets in *S. meliloti* and therefore whether their efflux would confer resistance.

BacA proteins are also critical for prolonged intracellular mammalian infections of *M. tuberculosis* and *B. abortus* [10],[11]. A *B. abortus* BacA-deficient mutant establishes an acute infection but was specifically defective upon entering the chronic phase of the infection in mice [11]. Mammalian hosts produce a cocktail of different AMPs depending upon the cell type [7]. The legume NCR peptides used in our in vitro experiments are cationic and defensin-like [5]. Mammalian defensins are primarily produced by epithelial cells and neutrophils as part of the innate immune response and invading pathogens encounter these early in their infection [7]. AMPs are also produced as part of the innate immune response by mammalian macrophages and pathogens such as *B. abortus* are challenged with these peptides within their phagosome compartments [29]. We showed previously that the *B. abortus* BacA protein complements the chronic infection defect of the *S. meliloti* BacA-deficient strain within *Medicago* legumes [23]. In this study, we were able to demonstrate that the *B. abortus* BacA protein complements the hypersensitivity of the *S. meliloti* BacA-deficient mutant towards the antimicrobial action of NCR247 in vitro. It is well established that bacterial protection against host AMPs is important for the establishment of acute bacterial pathogen-host infections [6]. For example, the two-component PhoPQ system protects *Salmonella enterica* serovar Typhimurium against AMPs and is essential for macrophage survival [30]. Our findings suggest that BacA-mediated protection of pathogens against host AMPs is also key for the establishment of chronic pathogen-host infections.

Since there are over three hundred different NCR peptides produced by *M. truncatula* root nodules [5], we took advantage of the *M. truncatula dnf1* mutant as a means to completely block NCR peptide trafficking into the symbiosome compartment (Figure 4B). However, since the *M. truncatula dnf1* mutant is defective in a nodule-specific signal peptidase complex [25], it could also be affected in transport of other proteins to the symbiosomes. Therefore, at this stage, we cannot formally rule out the possibility that survival of the *S. meliloti* BacA-deficient mutant in the *dnf1* mutant might be more complex than just the absence of NCRs and might also be due to the absence of one or more of these DNF1-dependent symbiosome proteins. We found, consistent with the published work [4],[25], that *S. meliloti* eventually dies in the *M. truncatula dnf1* mutant (Figure 4B). It is thought that the ultimate death of *S. meliloti* in this mutant legume might simply be due to a senescence process initiated in non-functional nodules [31]. However, it is also possible that the *S. meliloti* bacteroid features induced by the NCR peptides in the wild-type legume hosts are key for their ability to form prolonged infections in the host compartment. Therefore, since these bacterial changes would not occur in the *M. truncatula dnf1*, the bacteria could only form shorter term infections. Indeed, mammalian AMPs induce changes in bacterial pathogens permitting prolonged infections [32]. However, due to the complexity of AMP genes in mammalian hosts, the precise roles of these peptides on invading pathogens are not fully understood. For example, no such model system exists that can prevent the expression/trafficking of all mammalian AMPs. Future studies, blocking the trafficking of mammalian AMPs, in a similar way to the *M. truncatula dnf1* mutant, hold potential to uncover their precise role during mammalian infections.

In conclusion, we have shown that BacA plays a key role in the protection of *S. meliloti* against the antimicrobial action of host NCRs. Since there are over three hundred different NCR peptides and these peptides are expressed in different locations within the root nodules, the future study of different effects of the peptides on *S. meliloti* during the legume symbiosis has the potential to reveal further important findings into prolonged bacterial-host interactions.

## Materials and Methods

### Bacterial Growth

The sequenced *S. meliloti* Sm1021 strain (wild-type strain; formerly known as Rm1021) [33] and the SmGF1 BacA-deficient mutant [26] were used for the in vitro experiments. For the complementation, p*SmbacA* and p*BabacA* were used [23], whereby respectively the *S. meliloti* and *B. abortus bacA* genes were cloned into the broad host range vector pRF771 under the control of the *trp* promoter [34]. To obtain early exponential phase cultures, all strains were grown initially in LB medium [35] supplemented with 2.5 mM MgSO_4_ and 2.5 mM CaCl_2_ (LB/MC) and with 500 µg ml^−1^ streptomycin (5 µg ml^−1^ tetracycline was also added for plasmid-containing strains) for 48 h at 30°C, 200 rpm. The cultures were then diluted into fresh LB medium and grown overnight at 30°C, 200 rpm until an OD_600_  = 0.1 to 0.3. Stationary phase cultures were obtained by growth in LB/MC for 72 h at 30°C, 200 rpm. For solid media, 15 g of agar was added per liter of medium.

### Flow Cytometry

To examine the effect of NCR247 on the cell elongation and genome amplification, early exponential phase cultures were washed in 10 mM sodium phosphate buffer (pH 7.0), diluted to an OD_600_ of 0.1, treated with either 0, 2, or 4 µM of NCR247 peptide for 3 h at 30°C, washed in buffer, and then re-suspended in 50 µl of buffer supplemented with 1% (v/v) toluene. Stationary cultures were also fixed following the same procedure. All fixed cells were then stored at 4°C until required and then 450 µl of buffer containing 10 µg ml^−1^ PI was added. After 15 min incubation on ice, flow cytometric measurements were performed on a Becton-Dickinson LSR II flow cytometer. To assess the permeabilization of the bacteria by the NCR247 peptide, cells were incubated directly after washing in 500 µl of buffer containing 10 µg ml^−1^ PI and then immediately measured by flow cytometry. PI fluorescence was excited using a 488 nm laser and detected using a 610/20 band pass filter detecting emissions between 600 and 620 nm. 10,000 events were recorded per measurement. All experiments were performed at least in duplicate and the flow cytometry data shown are a representative data set.

### Colony Forming Ability

Exponential phase cultures were washed three times in 10 mM sodium phosphate buffer pH 7.0, diluted to an OD_600_ of 0.1, and treated with either 0, 20, or 30 µM of NCR peptide for 3 h at 30°C. The samples were serially diluted in buffer and 10 µl aliquots of each dilution spotted in triplicate on LB agar. Colony-forming units per ml were determined after 72 h at 30°C. The error bars represent the standard deviation from the mean of the CFU of the three aliquots and the significance was tested using the Student's unpaired *t* test for comparison of a single data pair and ANOVA followed by a Student Newman-Keuls post-test for pairwise multiple comparisons. The data were plotted as a percentage of the untreated control culture and the data sets shown are representative of at least two independent experiments.

### Gene Expression Analysis by RT-PCR

RNA and cDNA were prepared from duplicate Jemalong root samples, wild-type nodules, and nodules induced by the BacA-deficient mutant as described [5]. Semi-quantitative analysis of gene expression was performed by PCR using the primer pairs CTTTGCTTGGTGCTGTTTAGATGG and ATTCCAAAGGCGGCTGCATA for the constitutive gene Histone 3-like (accession number TC106487); CACGGGTATGCCATGTAAGA and CACAGTAGATGAATGGAAACCGTA for *NCR084*; CCATACCAACAGTGTCGCCG and CTTCACTTTCCAAGTTCCACCC for *NCR247*; TTTTAGGTGTCGTAAAGGCACA and TCTCAGGCTTAACCACAATGAA for *NCR035*; and TGGCTCAGTTTCTTCTCTTTGTT and ATGTGATGTCCCCTGGTTTC for *NCR001*.

### Cellular Localization of NCR Peptides

*M. truncatula* Jemalong was transformed with *Agrobacterium rhizogenes* strain ARqua1, carrying either a *NCR035-mCHERRY* fusion under the control of the *NCR035* promoter on plasmid pK7m34GW-rolD::EGFP-pNCR035:NCR035-cherryNOHDEL-T35S [4] or a *NCR084-mCHERRY* fusion under the control of the *NCR084* promoter on plasmid pK7m34GW-rolD::EGFP-pNCR084::NCR084-cherryNOHDEL-T35S. Nodulation on transgenic roots was induced with either *S. meliloti* Sm1021 (wild-type strain) or Sm8386 (BacA-deficient mutant) [8]. Green fluorescence due to the presence of a *rolD*::eGFP-ER reporter cassette on the binary vectors was used to select transgenic roots/nodules. 2- to 3-wk-old nodules were harvested and embedded in 6% (w/v) agarose. Nodule sections of 70 µm were prepared with a Leica VT1200S vibratome and stained in 1 µM SYTO13 solution in water for 5 min. Sections were removed from the staining solution and mounted in deionized water for microscopy observation. Images were acquired at 1024×1024 pixels resolution with a Leica confocal laser scanning microscope TCS SP2.

### In Situ Live/Dead Staining

Wild-type and *dnf1-1 M. truncatula* Jemalong were grown and inoculated with either the *S. meliloti* wild-type strain Sm1021 or BacA-deficient strain, Sm8386. 3-wk-old nodules were harvested and embedded in 6% (w/v) agarose. Nodule sections of 70 µm were prepared with a Leica VT1200S vibratome and incubated for 20 min in live/dead staining solution (5 µM SYTO9 and 30 µM PI in 50 mM Tris pH 7.0 buffer; Live/Dead BacLight, Invitrogen). Sections were removed from the staining solution and mounted in deionized water for microscopy observation. Images were acquired at 1024×1024 pixels resolution with a Leica confocal laser scanning microscope TCS SP2.

### NCR247 Synthesis

NCR247 was assembled on a peptide synthesizer (Applied Biosystem), using the stepwise solid phase Fmoc method on Fmoc-Rink-Amid AM resin on a 0.1 mmol scale. The N-9-Fluorenylmethoxycarbonyl amino acid used for the synthesis had the following side-chain protecting groups: trityl for cysteine at positions 1–2 and acetoamidomethyl for cysteine at positions 3–4, trityl for asparagine and glutamine, tert-butyl for aspartic acid, and 2,2,4,6,7,-pentamethyl-dihydrobenzofuran-5-sulfonyl for arginine. For each coupling step, the Fmoc-protected amino acid and coupling reagent were added in 5-fold molar excess with respect to resin substitution. Couplings were carried out with O-Benzotriazole-N,N,N′,N′-tetramethyl-uronium-hexafluoro-phosphate (HBTU). Cleavage from the resin and side chain de-protection of the synthesized peptides were carried out by treatment with a solution of 80% (v/v) trifluoroacetic acid, 5% (v/v) water, 5% (v/v) phenol, 5% (v/v) thioanisole, 2.5% (v/v) ethandithiol, and 2.5% (v/v) triisopropylsilane for 2 h. After precipitation with a cold 1∶1 mixture of tert-butylmethyether-petroleum ether and repeated washing with tert-butylmethyether-petroleum ether (1∶1), all peptides were purified to homogeneity by reversed-phase HPLC.

### NCR247 Folding

To create the disulfide bridge between the 1^st^ and 2^nd^ cysteine residues, a purified peptide solution was used from a semi-preparative column. A 1 M aqueous sodium hydroxide solution was added until a pH 7.5-7.8 was reached and then 1 M Tris-HCl buffer pH 8 was added until a 250 µM peptide concentration was obtained. Eight molar equivalents of a 0.1 M aqueous hydrogen peroxide solution were then added and the oxidative folding was followed by analytical RP-HPLC. After completion of the folding (30–60 min), the peptide solution was buffered to pH 2.2 with phosphoric acid and purified to homogeneity by semi-preparative RP-HPLC. To create the second disulfide bridge in the peptide between the 3^rd^ and 4^th^ cysteine residues, the purified peptide solution from a semi-preparative column was diluted with a 1∶1 mixture of water-methanol to reach a 150 µM final peptide concentration. An aqueous 0.1 M HCl solution (3 molar equivalents) and a 0.15 M solution of iodine in MeOH (5 molar equivalents) were added. The folding was followed by analytical RP-HPLC. After completion of the folding (60–120 min), the excess iodine was quenched by ascorbic acid (1 M in water) and the solvent was evaporated under vacuum. The peptides were then purified to homogeneity by semi-preparative RP-HPLC, analyzed by ESI-MS, and lyophilized.

### Reversed Phase HPLC

Analytical and semi-preparative Reversed Phase High Performance Liquid Chromatography (RP-HPLC) was carried out on a Tri Rotar-VI HPLC system equipped with a MD-910 multichannel detector for analytical purposes or with a Uvidec-100-VI variable UV detector for preparative purpose (all from JASCO, Tokyo, Japan). A Jupiter 5u C18 300A column (150×4.6 mm) was used for analytical runs and a Jupiter 10u C18 Proteo 90A (250×21.2 mm) for peptide purification. Crude peptides were purified by semi-preparative RP-HPLC. The column was eluted with a solvent system consisting of (1) 0.1% aqueous trifluoroacetic acid and (2) 0.7% of trifluoroacetic acid in 70% aqueous acetonitrile in a linear gradient mode for 40 min at 14 ml min^−1^ flow. The eluent was monitored at 220 nm. The fractions were checked by analytical RP-HPLC using a linear gradient elution with the two-solvent system described above at a flow rate of 1 ml min^−1^. The peaks were monitored at 220 nm.

### NCR035 Peptide Synthesis and Analysis

NCR035 was commercially synthesized (Innovagen, Lund, Sweden) and its purity and mass determined by HPLC and ESI-MS, respectively. RP-HPLC confirmed that the purified NCR035 peptide contained one peptide species with greater than 90% (w/v) purity. ESI-MS showed that the peptide had a mass of 3906.61 Da, consistent with the presence of two non-localized S-S bridges.

## Supporting Information

Figure S1 Mass spectrometry and HPLC analysis of NCR247 peptide. (A) ESI-MS of the NCR247 peptide. This analysis is in agreement with a peptide of 3004.5 Da and demonstrates the correct localization of the S-S bridges. (B) RP-HPLC analysis of the NCR247 peptide. The arrow indicates the single peptide peak with greater than 95% purity.
(TIF)

Figure S2 NCR247 peptide induces bacteroid-like features in cultured *S. meliloti*. Flow cytometry analysis of the in vitro *S. meliloti* wild-type strain (A–D) or the BacA-deficient mutant (E–H) with (shaded peaks, solid lines) and without (white peaks, dashed lines) 2 µM (A,B,E,F) or 4 µM NCR247 (C,D,G,H) exposure for 3 h. The forward scatter (FSC) was measured to estimate relative bacterial cell size (A,C,E,G) and the PI fluorescence was measured to estimate the relative bacterial DNA content (B,D,F,H).
(TIF)

Figure S3 BacA proteins protect *S. meliloti* in vitro against the antimicrobial activity of NCR035. (A) NCR035 peptide sequence with the conserved cysteine residues in bold and underlined. (B) Colony forming ability of the indicated strains was assessed after exposure towards 30 µM NCR035 peptide for 3 h. Bars represent mean ± SD. The significance value **p≤0.01 was determined using the two-sided Student's unpaired *t* test and results are representative for two independent experiments.
(TIF)

Figure S4 The hypersensitivity of the BacA-deficient *S. meliloti* mutant towards NCR247 is complemented by reintroduction of the *S. meliloti bacA* gene or the *B. abortus bacA* gene. Colony forming ability in the indicated strains was assessed after exposure towards 20 µM of the NCR247 peptide for 3 h. Control vector (pRF771), wild-type *S. meliloti bacA* gene cloned into pRF771 (p*SmbacA*), and wild-type *B. abortus bacA* gene cloned into pRF771 (p*BabacA*). Bars represent mean ± SD. The significance value ***p≤0.001 was determined using ANOVA followed by a Student Newman-Keuls post-test and results are representative for at least two independent experiments.
(TIF)

Figure S5 RT-PCR analysis of *NCR* gene expression. Expression analysis of the indicated *NCR* genes in *M. truncatula* wild-type roots and nodules induced by either the *S. meliloti* wild-type strain or the BacA-deficient mutant. Histone 3-like (H3 like) is used as a constitutive control.
(TIF)

Figure S6 NCR035 and NCR084 are transported to symbiosomes, even in the absence of bacterial BacA. Confocal microscopy of the *S. meliloti* wild-type (A,C,E) and BacA-deficient mutant (B,D,F) strains infected transgenic nodules expressing NCR035-mCHERRY under the control of the *NCR035* promoter (A–D) or NCR084-mCHERRY under the control of the *NCR084* promoter (E–F). Whole nodule transversal sections are shown in (A,B) and symbiotic nodule cells in (C–F). In wild-type strain-infected nodules, NCR035 or NCR084 co-localize with bacteroids. NCRs also co-localize with the rhizobia in the BacA-deficient mutant infected cells, but a proportion of the peptides are secreted to the outside of the nodule cells. The green fluorescence in (A,B) originates from the *rolD*::eGFP-ER reporter cassette which is present on the binary vector and used to select transgenic roots/nodules. Scale bars are 100 µm (A,B) or 10 µm (C–F).
(TIF)

Figure S7 The *S. meliloti* BacA-deficient mutant undergoes rapid death in symbiosomes. Confocal microscopy of either the *S. meliloti* wild-type strain-infected nodules (A,C) or the BacA-deficient mutant-infected nodules (B,D) from wild-type *M. truncatula* (A,B) and from an *M. truncatula dnf1* mutant (C,D) stained with a mixture of SYTO9 (green signal) and PI (red signal). Live bacterial cells are stained by SYTO9 and dead bacteria are stained by PI. The nodule meristem is indicated by an asterisk. The strong red PI staining in the meristem is resulting from the staining of plant nuclei. Scale bars are 50 µm.
(TIF)

Figure S8 Viability of symbiosome bacteria in the *M. truncatula dnf-1* mutant nodules. (A–C) Confocal microscopy of *S. meliloti* wild-type-infected *dnf1* nodules stained with a mixture of SYTO9 (green signal) and PI (red signal). (C) Enlargement of the image in (B). These nodules have an extended zone with symbiotic cells containing live bacteria stained with SYTO9 (green) (double asterisk). In older, root-proximal zones of the nodule (triple asterisk), the bacteria are dead as revealed by their red PI staining most likely reflecting senescence of the cells. The nodule meristem is indicated by a single asterisk. The strong red PI staining in the meristem in (A) is resulting from the staining of plant nuclei. Scale bars are 50 µm.
(TIF)

Table S1 Correlation between NCR gene expression in nodules, requirement of BacA, and bacteroid type. ^1^ Reported presence (yes) or absence (no) of NCR gene expression in nodules. ^2^ Requirement of BacA protein for efficient, nitrogen-fixing symbiosis; nd, no data available. ^3^ Mergaert et al., 2006 [3] and unpublished data.
(DOC)

## Abbreviations

NCR: nodule-specific cysteine-rich
PI: propidium iodide
