# Supplementary figures and images for: Protection of Sinorhizobium against Host Cysteine-Rich Antimicrobial Peptides Is Critical for Symbiosis
Source: PLoS Biol. 2011 Oct 4;9(10):e1001169. doi: 10.1371/journal.pbio.1001169 (PMC3186793; doi:10.1371/journal.pbio.1001169)

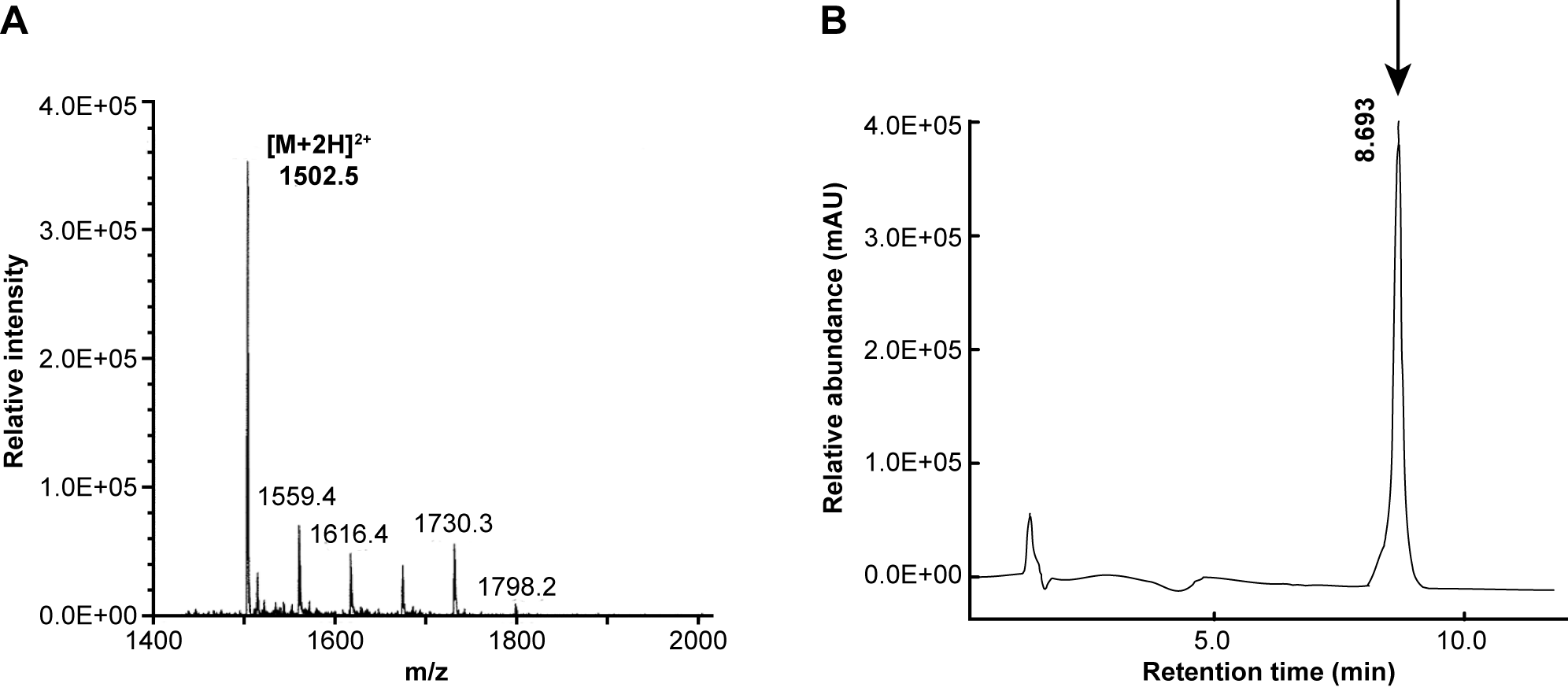

Supplement: Figure S1 — Mass spectrometry and HPLC analysis of NCR247 peptide. (A) ESI-MS of the NCR247 peptide. This analysis is in agreement with a peptide of 3004.5 Da and demonstrates the correct localization of the S-S bridges. (B) RP-HPLC analysis of the NCR247 peptide. The arrow indicates the single peptide peak with greater than 95% purity. (TIF) [file pbio.1001169.s001.tif]

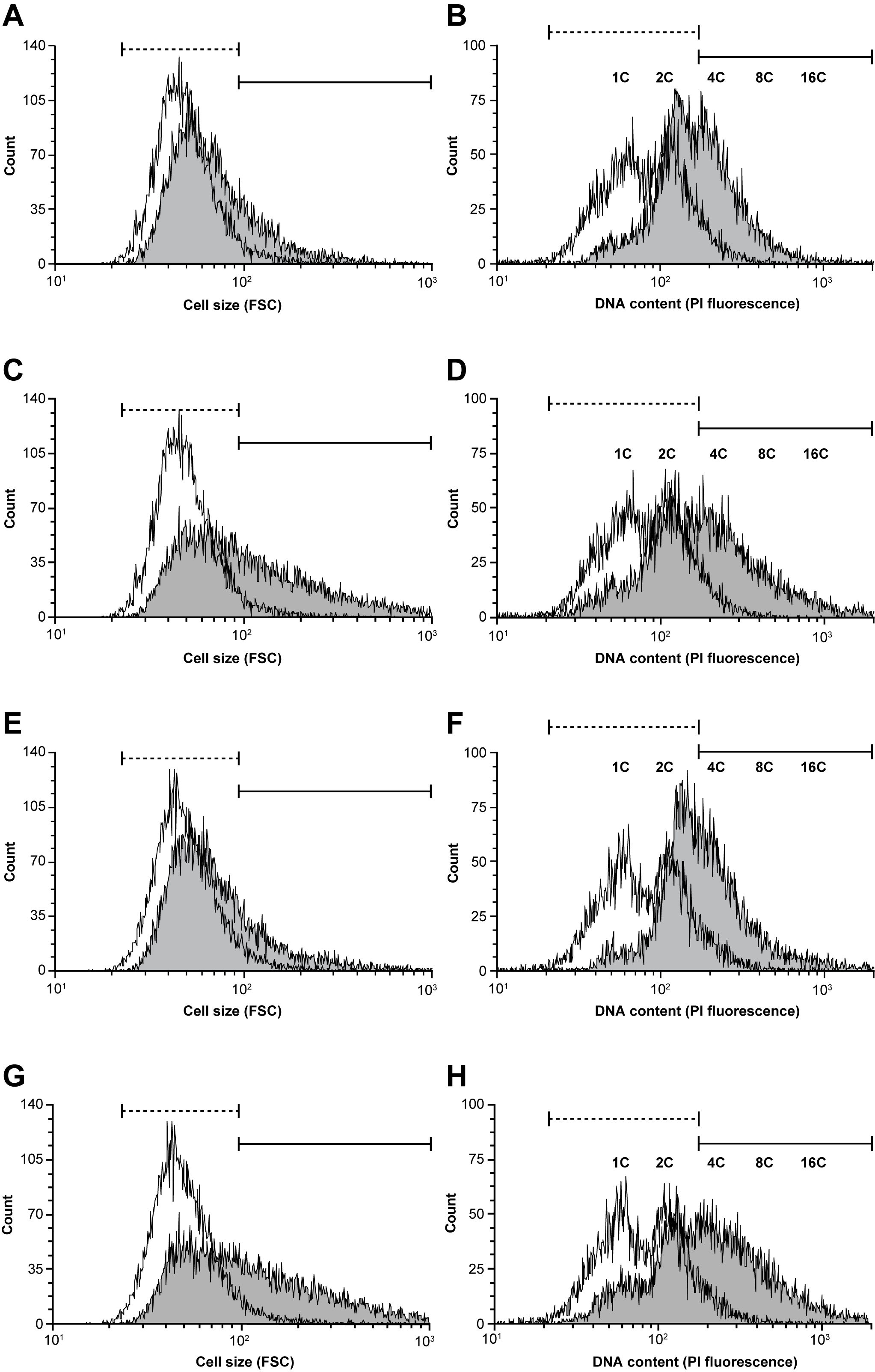

Supplement: Figure S2 — NCR247 peptide induces bacteroid-like features in cultured S. meliloti. Flow cytometry analysis of the in vitro S. meliloti wild-type strain (A–D) or the BacA-deficient mutant (E–H) with (shaded peaks, solid lines) and without (white peaks, dashed lines) 2 µM (A,B,E,F) or 4 µM NCR247 (C,D,G,H) exposure for 3 h. The forward scatter (FSC) was measured to estimate relative bacterial cell size (A,C,E,G) and the PI fluorescence was measured to estimate the relative bacterial DNA content (B,D,F,H). (TIF) [file pbio.1001169.s002.tif]

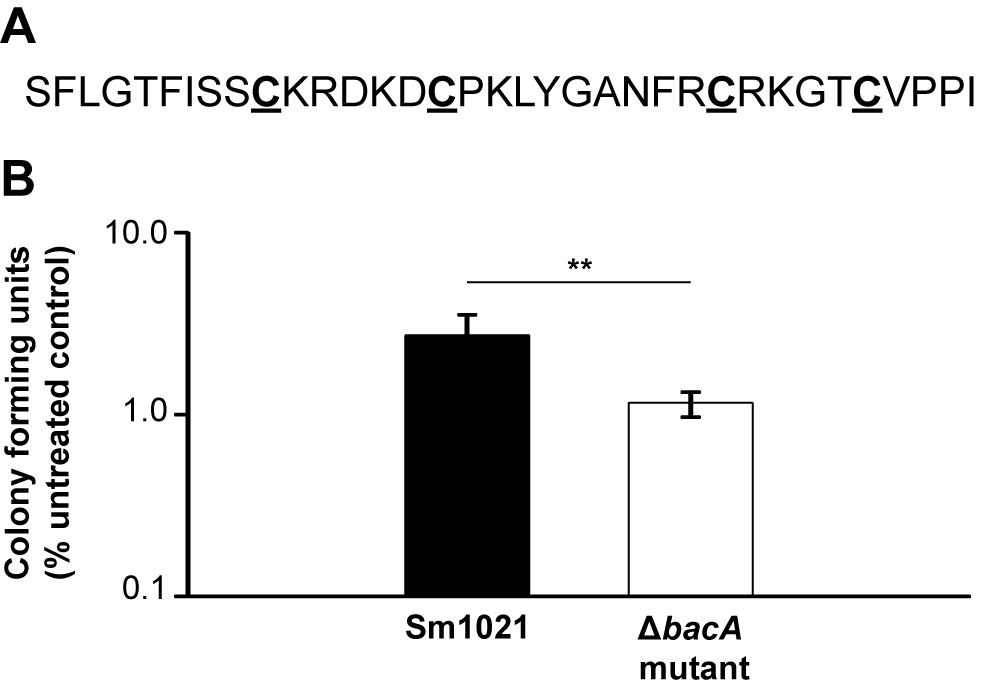

Supplement: Figure S3 — BacA proteins protect S. meliloti in vitro against the antimicrobial activity of NCR035. (A) NCR035 peptide sequence with the conserved cysteine residues in bold and underlined. (B) Colony forming ability of the indicated strains was assessed after exposure towards 30 µM NCR035 peptide for 3 h. Bars represent mean ± SD. The significance value **p≤0.01 was determined using the two-sided Student's unpaired t test and results are representative for two independent experiments. (TIF) [file pbio.1001169.s003.tif]

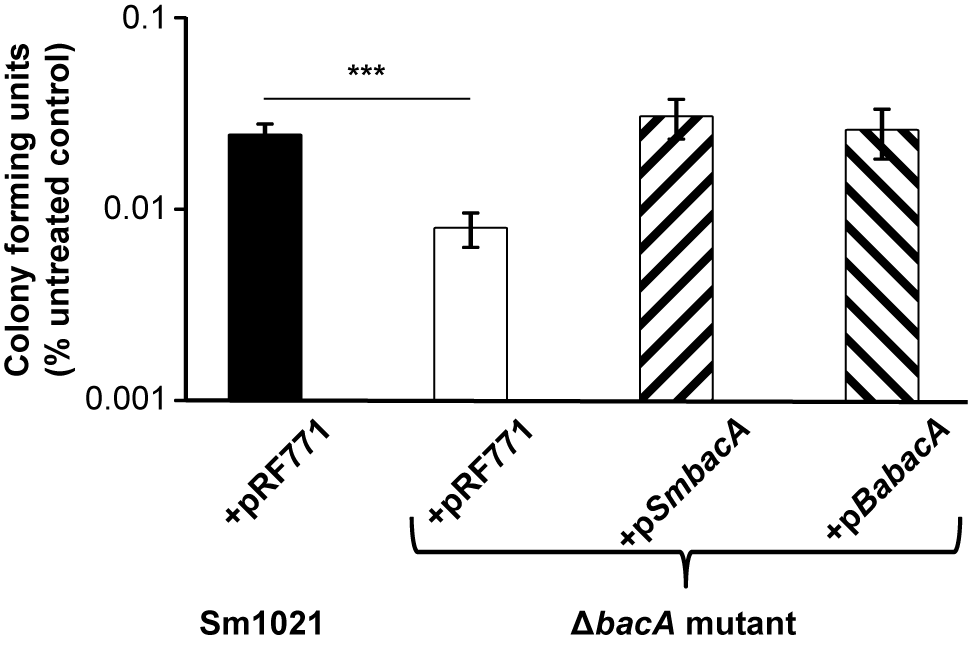

Supplement: Figure S4 — The hypersensitivity of the BacA-deficient S. meliloti mutant towards NCR247 is complemented by reintroduction of the S. meliloti bacA gene or the B. abortus bacA gene. Colony forming ability in the indicated strains was assessed after exposure towards 20 µM of the NCR247 peptide for 3 h. Control vector (pRF771), wild-type S. meliloti bacA gene cloned into pRF771 (pSmbacA), and wild-type B. abortus bacA gene cloned into pRF771 (pBabacA). Bars represent mean ± SD. The significance value ***p≤0.001 was determined using ANOVA followed by a Student Newman-Keuls post-test and results are representative for at least two independent experiments. (TIF) [file pbio.1001169.s004.tif]

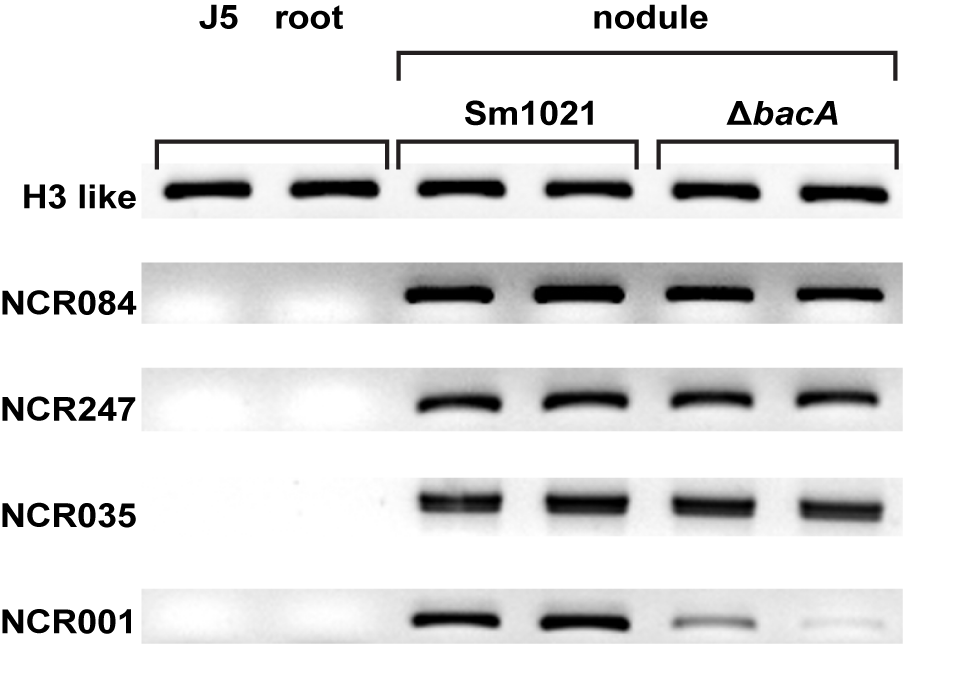

Supplement: Figure S5 — RT-PCR analysis of NCR gene expression. Expression analysis of the indicated NCR genes in M. truncatula wild-type roots and nodules induced by either the S. meliloti wild-type strain or the BacA-deficient mutant. Histone 3-like (H3 like) is used as a constitutive control. (TIF) [file pbio.1001169.s005.tif]

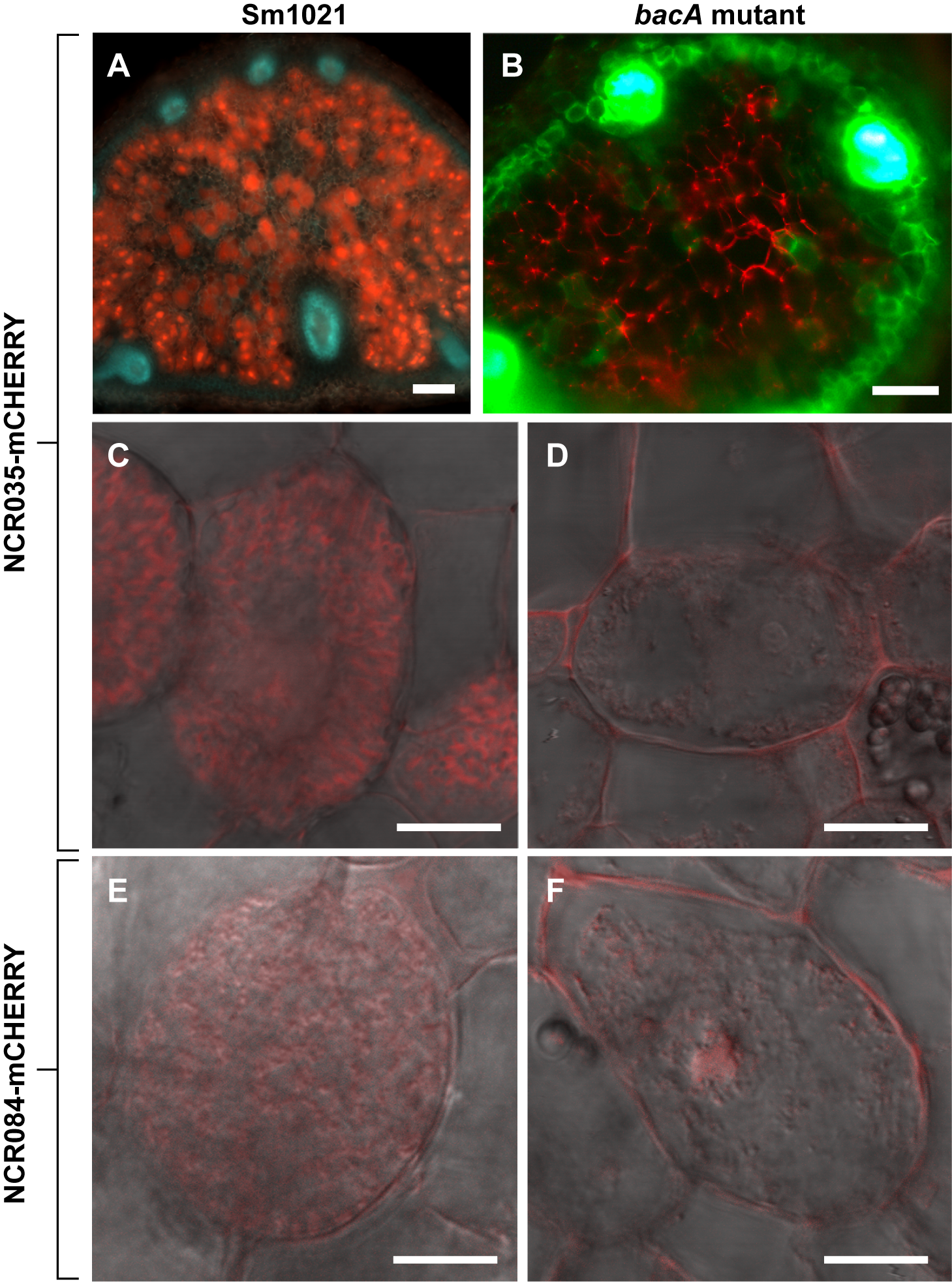

Supplement: Figure S6 — NCR035 and NCR084 are transported to symbiosomes, even in the absence of bacterial BacA. Confocal microscopy of the S. meliloti wild-type (A,C,E) and BacA-deficient mutant (B,D,F) strains infected transgenic nodules expressing NCR035-mCHERRY under the control of the NCR035 promoter (A–D) or NCR084-mCHERRY under the control of the NCR084 promoter (E–F). Whole nodule transversal sections are shown in (A,B) and symbiotic nodule cells in (C–F). In wild-type strain-infected nodules, NCR035 or NCR084 co-localize with bacteroids. NCRs also co-localize with the rhizobia in the BacA-deficient mutant infected cells, but a proportion of the peptides are secreted to the outside of the nodule cells. The green fluorescence in (A,B) originates from the rolD::eGFP-ER reporter cassette which is present on the binary vector and used to select transgenic roots/nodules. Scale bars are 100 µm (A,B) or 10 µm (C–F). (TIF) [file pbio.1001169.s006.tif]

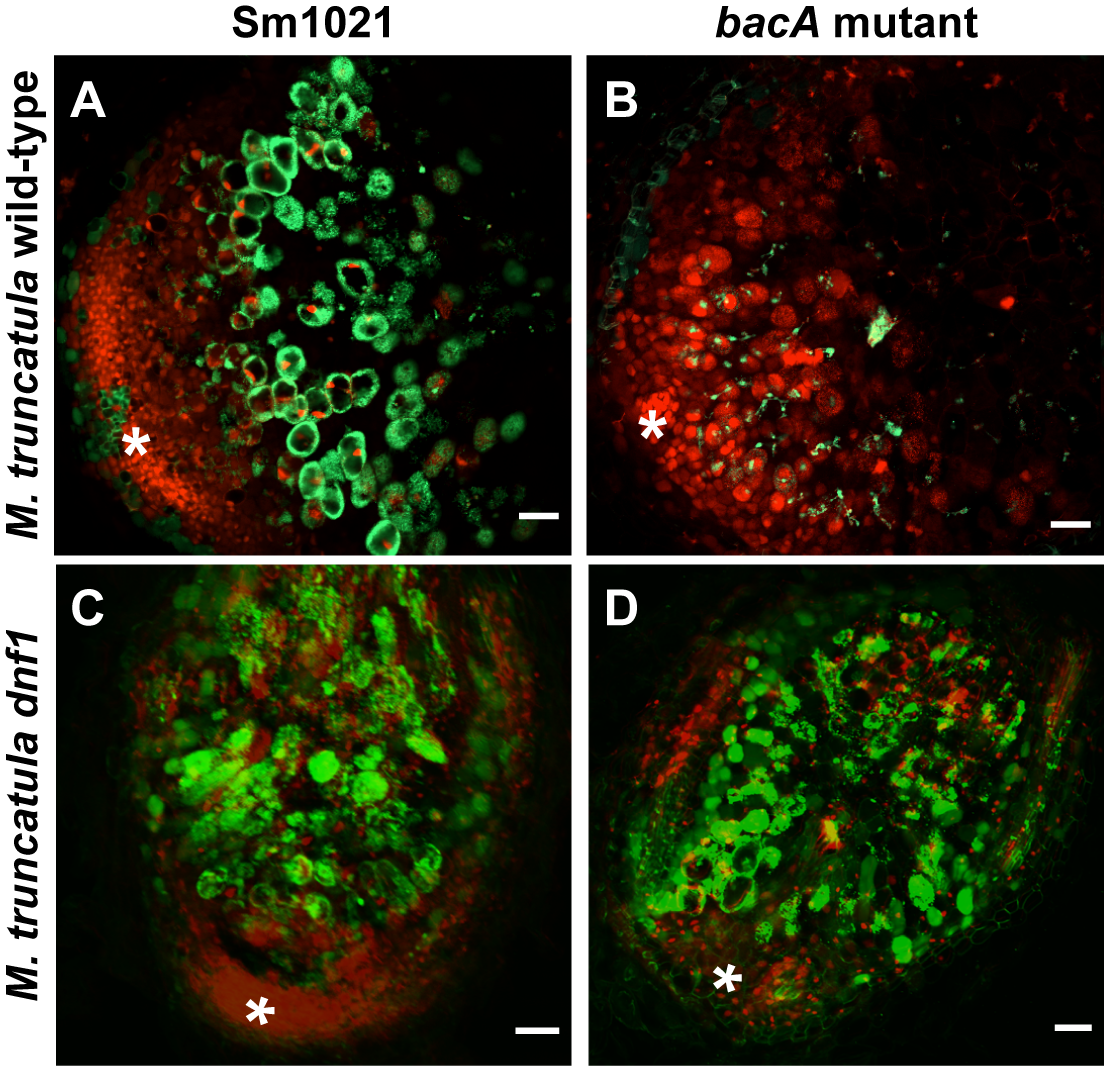

Supplement: Figure S7 — The S. meliloti BacA-deficient mutant undergoes rapid death in symbiosomes. Confocal microscopy of either the S. meliloti wild-type strain-infected nodules (A,C) or the BacA-deficient mutant-infected nodules (B,D) from wild-type M. truncatula (A,B) and from an M. truncatula dnf1 mutant (C,D) stained with a mixture of SYTO9 (green signal) and PI (red signal). Live bacterial cells are stained by SYTO9 and dead bacteria are stained by PI. The nodule meristem is indicated by an asterisk. The strong red PI staining in the meristem is resulting from the staining of plant nuclei. Scale bars are 50 µm. (TIF) [file pbio.1001169.s007.tif]

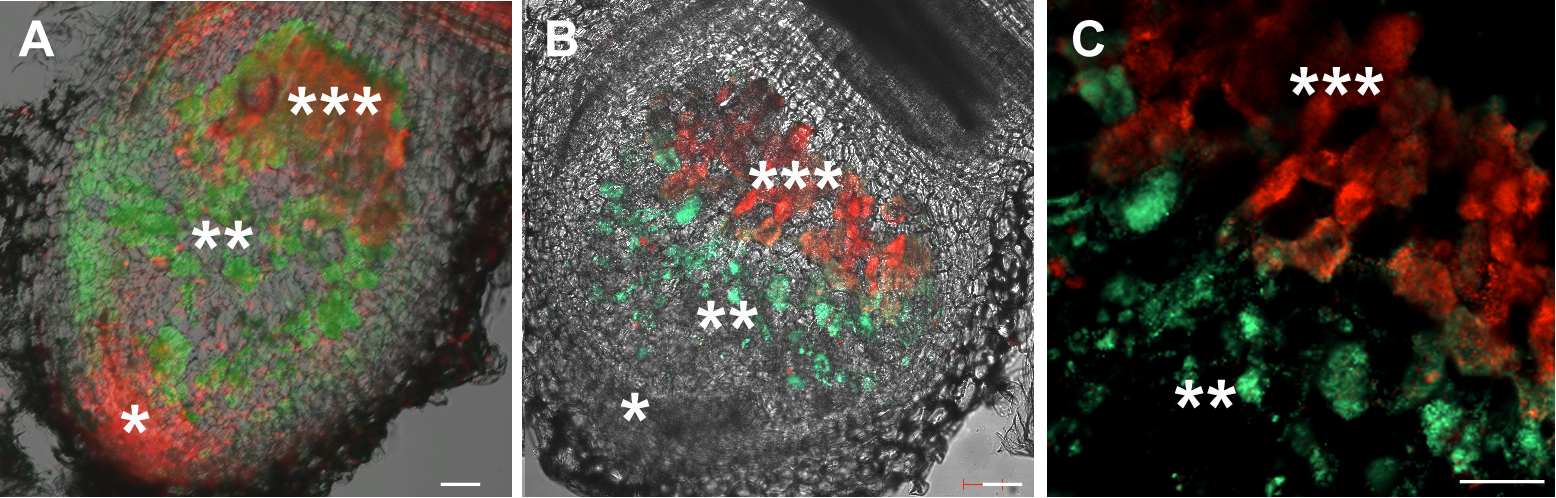

Supplement: Figure S8 — Viability of symbiosome bacteria in the M. truncatula dnf-1 mutant nodules. (A–C) Confocal microscopy of S. meliloti wild-type-infected dnf1 nodules stained with a mixture of SYTO9 (green signal) and PI (red signal). (C) Enlargement of the image in (B). These nodules have an extended zone with symbiotic cells containing live bacteria stained with SYTO9 (green) (double asterisk). In older, root-proximal zones of the nodule (triple asterisk), the bacteria are dead as revealed by their red PI staining most likely reflecting senescence of the cells. The nodule meristem is indicated by a single asterisk. The strong red PI staining in the meristem in (A) is resulting from the staining of plant nuclei. Scale bars are 50 µm. (TIF) [file pbio.1001169.s008.tif]
