# Supplementary material for: Protection of Sinorhizobium against Host Cysteine-Rich Antimicrobial Peptides Is Critical for Symbiosis
Source: PLoS Biol. 2011 Oct 4;9(10):e1001169. doi: 10.1371/journal.pbio.1001169 (PMC3186793; doi:10.1371/journal.pbio.1001169)
Supplement: Table S1 — Correlation between NCR gene expression in nodules, requirement of BacA, and bacteroid type. 1 Reported presence (yes) or absence (no) of NCR gene expression in nodules. 2 Requirement of BacA protein for efficient, nitrogen-fixing symbiosis; nd, no data available. 3 Mergaert et al., 2006 [3] and unpublished data. (DOC) [file pbio.1001169.s009.doc]

Table S1 Correlation between NCR gene expression in nodules, requirement of BacA and bacteroid type.

| **Legume species** | **NCRs^1^** | **Source** | **Rhizobial symbiont** | **BacA*^2^*** | **Source** | **Bacteroid type^3^** |
| --- | --- | --- | --- | --- | --- | --- |
| *Phaseolus vulgaris* (bean) | no | <http://compbio.dfci.harvard.edu/tgi/> | *Rhizobium leguminosarum* bv. *phaseoli* | no |  | reversible |
|  |  |  | *Rhizobium etli* | no |  |  |
| *Vigna unguiculata* (cowpea) | no | <http://compbio.dfci.harvard.edu/tgi/> | *Sinorhizobium* NGR234 | no |  | reversible |
| *Lotus japonicus* | no |  | *Mesorhizobium loti* | no |  | reversible |
| *Glycine max* (soybean) | no |  | *Bradyrhizobium japonicum* | nd |  | reversible |
|  |  |  | *Sinorhizobium fredii* HH103 | nd |  |  |
| *Medicago truncatula* | yes |  | *Sinorhizobium meliloti* Sm1021 | yes |  | terminal |
| *Medicago sativa* (alfalfa) | yes |  | *Sinorhizobium meliloti* Sm1021 | yes |  | terminal |
| *Pisum sativum* (pea) | yes |  | *Rhizobium leguminosarum* bv. *viciae* | yes |  | terminal |
| *Astragalus sinicus* | yes |  | *Mesorhizobium huakuii* | yes |  | terminal |
| *Trifolium repens* (white clover) | yes |  | *Rhizobium leguminosarum* bv. *trifolii* | nd |  | terminal |
| *Galega orientalis* (goat’s rue) | yes |  | *Rhizobium galegae* | nd |  | terminal |
| *Vicia faba* (broad bean) | yes |  | *Rhizobium leguminosarum* bv. *viciae* | nd |  | terminal |

^1^ reported presence (yes) or absence (no) of NCR gene expression in nodules.

^2^ requirement of BacA protein for efficient, nitrogen-fixing symbiosis; nd: no data available.

^3^ Mergaert et al., 2006 [3] and data not shown.

**References**

1. Karunakaran R, Haag AF, East AK, Ramachandran VK, Prell J, et al. (2010) BacA is essential for bacteroid development in nodules of galegoid, but not phaseoloid, legumes. J Bacteriol 192: 2920-2928.

2. Ardissone S, Kobayashi H, Kambara K, Rummel C, Noel KD, et al. (2011) Role of BacA in lipopolysaccharide synthesis, peptide transport and nodulation by *Rhizobium* sp. NGR234. J Bacteriol: 2218–2228.

3. Alunni B, Kevei Z, Redondo-Nieto M, Kondorosi A, Mergaert P, et al. (2007) Genomic organization and evolutionary insights on *GRP* and *NCR* genes, two large nodule-specific gene families in *Medicago truncatula*. Mol Plant Microbe Interact 20: 1138-1148.

4. Maruya J, Saeki K (2010) The *bacA* gene homolog, mlr7400, in *Mesorhizobium loti* MAFF303099 is dispensable for symbiosis with *Lotus japonicus* but partially capable of supporting the symbiotic function of *bacA* in *Sinorhizobium meliloti*. Plant Cell Physiol 51: 1443-1452.

5. Mergaert P, Nikovics K, Kelemen Z, Maunoury N, Vaubert D, et al. (2003) A novel family in *Medicago truncatula* consisting of more than 300 nodule-specific genes coding for small, secreted polypeptides with conserved cysteine motifs. Plant Physiol 132: 161-173.

6. Graham MA, Silverstein KA, Cannon SB, VandenBosch KA (2004) Computational identification and characterization of novel genes from legumes. Plant Physiol 135: 1179-1197.

7. Maunoury N, Redondo-Nieto M, Bourcy M, Van De Velde W, Alunni B, et al. (2010) Differentiation of symbiotic cells and endosymbionts in *Medicago truncatula* nodulation are coupled to two transcriptome-switches. PLoS ONE 5: e9519.

8. Jimenez-Zurdo JI, Frugier F, Crespi MD, Kondorosi A (2000) Expression profiles of 22 novel molecular markers for organogenetic pathways acting in alfalfa nodule development. Mol Plant Microbe Interact 13: 96-106.

9. Glazebrook J, Ichige A, Walker GC (1993) A *Rhizobium meliloti* homolog of the *Escherichia coli* peptide-antibiotic transport protein SbmA is essential for bacteroid development. Genes Dev 7: 1485-1497.

10. Scheres B, van Engelen F, van der Knaap E, van de Wiel C, van Kammen A, et al. (1990) Sequential induction of nodulin gene expression in the developing pea nodule. Plant Cell 2: 687-700.

11. Kato T, Kawashima K, Miwa M, Mimura Y, Tamaoki M, et al. (2002) Expression of genes encoding late nodulins characterized by a putative signal peptide and conserved cysteine residues is reduced in ineffective pea nodules. Mol Plant Microbe Interact 15: 129-137.

12. Chou MX, Wei XY, Chen DS, Zhou JC (2006) Thirteen nodule-specific or nodule-enhanced genes encoding products homologous to cysteine cluster proteins or plant lipid transfer proteins are identified in *Astragalus sinicus* L. by suppressive subtractive hybridization. J Exp Bot 57: 2673-2685.

13. Tan XJ, Cheng Y, Li YX, Li YG, Zhou JC (2009) BacA is indispensable for successful *Mesorhizobium-Astragalus* symbiosis. Appl Microbiol Biotechnol 84: 519-526.

14. Crockard A, Bjourson J, Dazzo B, Cooper JE (2002) A white clover nodulin gene, *dd23b*, encoding a cysteine cluster protein, is expressed in roots during the very early stages of interaction with *Rhizobium leguminosarum* biovar *trifolii* and after treatment with chitolipooligosaccharide Nod factors. J Plant Res 115: 439-447.

15. Kaijalainen S, Schroda M, Lindstrom K (2002) Cloning of nodule-specific cDNAs of *Galega orientalis*. Physiol Plant 114: 588-593.

16. Frühling M, Albus U, Hohnjec N, Geise G, Pühler A, et al. (2000) A small gene family of broad bean codes for late nodulins containing conserved cysteine clusters. Plant Sci 152: 67-77.
